# Anti-Allergic Rhinitis Effects of Medicinal Plants and Their Bioactive Metabolites *via* Suppression of the Immune System: A Mechanistic Review

**DOI:** 10.3389/fphar.2021.660083

**Published:** 2021-04-13

**Authors:** Nur Amira Rahim, Ibrahim Jantan, Mazlina Mohd Said, Juriyati Jalil, Amirul Faiz Abd Razak, Khairana Husain

**Affiliations:** ^1^ Drug and Herbal Research Centre, Faculty of Pharmacy, Universiti Kebangsaan Malaysia, Kuala Lumpur, Malaysia; ^2^ Institute of Systems Biology, Universiti Kebangsaan Malaysia, Bangi, Malaysia

**Keywords:** medicinal plants, anti-allergic rhinitis, immune system, immunoglobulin E, molecular mechanisms, toxicology

## Abstract

Allergic rhinitis (AR) is a common inflammatory condition of the nasal mucosa and it is an immunoglobulin E–mediated disease. The incidence and prevalence of AR globally have been escalating over recent years. Antihistamines, intranasal corticosteroids, decongestants, intranasal anticholinergics, intranasal cromolyn, leukotriene receptor antagonists and immunotherapy have been used in the treatment of AR. However, there is a need to search for more effective and safer remedies as many of the current treatments have reported side effects. Medicinal plants have been used traditionally to relief symptoms of AR but their efficacy and safety have not been scientifically proven. In this review, up-to-date reports of studies on the anti-allergic rhinitis of several medicinal plants and their bioactive metabolites through suppression of the immune system are compiled and critically analyzed. The plant samples were reported to suppress the productions of immunoglobulin E, cytokines and eosinophils and inhibit histamine release. The suppression of cytokines production was found to be the main mechanistic effect of the plants to give symptomatic relief. The prospect of these medicinal plants as sources of lead molecules for development of therapeutic agents to treat AR is highlighted. Several bioactive metabolites of the plants including shikonin, okicamelliaside, warifteine, methylwarifteine, luteolin-7-*O*-rutinoside, tussilagone, petasin, and mangiferin have been identified as potential candidates for development into anti-allergic rhinitis agents. The data collection was mainly from English language articles published in journals, or studies from EBSCOHOST, Medline and Ovid, Scopus, Springer, and Google Scholar databases from the year 1985–2020. The terms or keywords used to find relevant studies were allergic rhinitis OR pollinosis OR hay fever, AND medicinal plant OR single plant OR single herb OR phytotherapy. This comprehensive review serves as a useful resource for medicinal plants with anti-allergic rhinitis potential, understanding the underlying mechanisms of action and for future exploration to find natural product candidates in the development of novel anti-allergic rhinitis agents.

## Introduction

Allergic rhinitis (AR) is one of the common allergic diseases affecting 30% of the world population (Tohidinik et al., 2019). It is also known as hay fever, typically causes symptoms of runny nose, clear nasal discharge, sneezing nasal pruritus and airflow obstruction caused by immunoglobulin E (IgE)-mediated reactions. AR is triggered by potential allergens and involves mucosal inflammation driven by type 2 helper T (Th2) cells (Wheatley and Togias, 2015). AR is one of the types of allergy that manifest an abnormal regulation of the immune system. Epidemiological studies have revealed that the prevalence of AR has increased progressively in more developed countries, and currently affects 10–40% of adults and 2–25% of children (Zhang and Zhang, 2019). It is one of the common types of allergic condition which might not appear to be serious as it is not associated with severe morbidity and mortality but affects the quality of life. The allergic disease may also substantially cause an economic burden to the patient due to inadequate management of the allergic disease (Zuberbier et al., 2014). Thus, the needs on medication and alternative therapy to alleviate the symptoms are very crucial. There are some diseases particularly allergic reactions that have been found to be associated with AR. The prevalence of AR has shown an increasing pattern in a similar fashion to that of asthma (Asher et al., 2006; Tohidinik et al., 2019). It was found that eczema in infancy might have a causal effect on AR in children with and perhaps without asthma (Hopper et al., 2012). There is a close epidemiologic relationship of allergic conjunctivitis with AR (Bielory, 2010). Genetic factor might be one of the etiologies of AR as a study has shown that having a parent with AR may double the risk (Westman et al., 2013). Seeing that it might be leading to development of other diseases, this health issue requires great involvement of patients and healthcare professionals.

The pathophysiology of AR disease is quite complex as it involves several chemical mediators or antibodies in immunological cascades. Therefore, these different mediators or antibodies involved in the pathophysiology of the disease might be the potential targets in treating AR. It is very crucial to understand the pathophysiology of this disease in developing a potential remedy and alternative therapy for a wide population of AR patients. Avoidance of potential allergens that might trigger an allergic reaction is the best way to prevent AR occurrence (Okubo et al., 2017). Allergens that could trigger the symptoms in AR include animal dander, dust mite, seafood as well as cigarette smoke. Heavy maternal cigarette smoking may increase the risk of having this disease in children in the first year of life (Skoner, 2001). Treating the symptoms by using conventional or alternative therapy may be efficient but it is important to identify the triggers or allergens to avoid repetitive occurrence. It may be difficult to avoid the potential allergen exposure and therefore the use of medicine is needed to provide fast relief. The use of conventional medicine has an increasing trend as the number of people having this disease has also increased.

Current treatment to treat symptoms of AR include the use of antihistamines, intranasal corticosteroids, decongestants, intranasal cromolyn, intranasal anticholinergics, leukotriene receptor antagonists and immunotherapy. Antihistamines inhibit receptor activity, and some can additionally stabilize the H_1_-receptor inverse agonists or mast cells (Leurs et al., 2002; Bousquet et al., 2008). The use of first-generation antihistamines has reported several side effects such as mental impairment, sedation and anticholinergic side effects (Bousquet et al., 2008). Antihistamine monotherapy has been reported to provide satisfactory relief of symptoms in some patients. However, for the relief of nasal congestion, antihistamines use is less effective and usually need to be combined with a decongestant or intranasal corticosteroid (May and Dolen, 2017). Intranasal corticosteroids decrease the inflammatory cytokines and mediators release, which help to reduce nasal mucosal inflammation that can reach maximum benefit within two weeks (Weiner et al., 1998). Corticosteroids administered by any route, including the intranasal route can cause adverse effects on the eyes, bones, the hypothalamic-pituitary-adrenal axis, and also local side effects such as candidiasis (Bielory, 2010). The side effects of the use of conventional medicines have led to many studies to find alternative ways to help to reduce the symptoms of this disease. Some natural products such as garlic and Andrographis paniculata (Burm.f.) Nees have been used to provide symptom relief for nasal congestion which is also one of the symptoms in AR. Various treatment options are targeting different key components to help to relieve AR symptoms. However, the scientific or evidence-based research on the efficacy and safety of the plants have not yet been proven in many studies upon AR models or subjects. Therefore, many studies have been conducted in search of medicinal plants that have a promising potential of being a better alternative to provide symptoms relief in AR.

The search for new therapeutic agents from medicinal plants to treat AR is definitely needed especially plants which have shown positive outcomes in exerting anti-allergic properties, particularly toward AR animal models. Medicinal plants and their bioactive metabolites possess different mechanisms of action in combating the mediators or immune system involved in AR inflammatory cascades or allergic reaction pathways. Some of these phytochemicals have anti-allergic properties as well as anti-inflammatory properties which may be useful to treat different types of allergic diseases and symptoms. Numerous studies have been performed to determine the anti-inflammatory and anti-allergic properties of medicinal plants particularly in treating AR through *in vivo* studies and clinical trials by using AR-induced model or AR patients, respectively. There are numerous number of mediators involved in the pathophysiology of AR. These different targeted cells or mediators are important key components for different types of phytochemicals found in medicinal plants. AR is one of the types of allergy that manifest an abnormal regulation of the immune system.

## Methods

The databases employed for data collection are mainly from EBSCOHOST, Medline and Ovid, Scopus, Springer, and Google Scholar databases from 1985 to 2020. The keywords used during searching include allergic rhinitis OR pollinosis OR hay fever, AND medicinal plant OR single plant OR single herb OR phytotherapy. Studies included in this mechanistic review were *in vivo, in vitro*, and clinical studies on medicinal plants that showed significant suppressive effect on AR. The sources of medicinal plants were verified, and only studies on single medicinal plant were considered. Table 1 shows a list of medicinal plants and phytochemicals which exhibited anti-allergic rhinitis effect *in vivo* and *in vitro* studies while Table 2 is a list of plants that have been investigated for anti-allergic rhinitis activity in clinical trials. Studies that were excluded from the searching and data extraction were polyherbal formulations, used as an adjunct therapy or as supplementation only and studies that were not using AR-induced model in animal studies or AR patients for its clinical studies. In addition, toxicological data on the medicinal plants that showed strong anti-allergic rhinitis effect were also gathered to discuss their safety levels for possible use in treating AR.

**TABLE 1:** Plants with anti-allergic rhinitis effects *in vivo* and *in vitro* studies.

Plant name	Family	Plant part used	Isolated compound/extract used	Assay type/Type of model or cells	Mechanism of action	References
*Acanthus ilicifolius* L	Acanthaceae	Ariel	95% ethanol extract	*In vivo*	Decreased the expression of leukocytes, eosinophils lymphocytes, neutrophils, monocytes and basophils	Sardar et al. (2018)
				Blood from toluene 2, 4-diisocyanate (TDI)-induced allergic mice model		
*Allium hookeri* thwaites	Amarylidaceae	Not stated	Ethyl alcohol extract	*In vivo*	It reduced IL-4, VEGF expression and inhibited TNFα in nasal mucosa tissue	Kim et al. (2019)
				Stimulated human mast cell line (HMC) 1 cells, and nasal mucosa of ovalbumin (OVA) sensitized mouse model of AR		
*Wurfbainia villosa* var. *xanthioides* (Wall. ex Baker) Skornick. and A.D.Poulsen	Zingiberaceae	Fruit	Aqueous extract	*In vivo*	Reduced histamine release, IgE and calcium ionophore-mediated expression of TNF-α	Kim et al. (2007)
				Blood samples from heart was taken for histamine serum measurement from male mice		
*Ampelopsis glandulosa* var. *brevipedunculata* (Maxim.) Momiy	Vitaceae	Fruit	Hot water extract	*In vivo*	Reduced H1R and IL-9 gene expression in the nasal mucosa	Islam et al. (2018)
				Nasal mucosa and protein serum from toluene-2,4diisocyanate (TDI)-sensitized rats		
*Asarum sieboldii* Miq	Aristolochiaceae	Root	Essential oil	*In vivo*	Reduced IgE and histamine levels. Reduced IL-17 and increased IFN-γ levels	Zhang and Kang (2020)
				Blood and serum sample from fundus venous plexus and nasal mucosa of OVA induced sixty male Sprague-Dawley rats		
*Bupleurum chinense* DC.	Apiaceae	Not stated	Dissolved saline *Bupleurum chinense* DC extract powder	*In vivo*	It suppressed mast cell infiltration and down-regulated IL-4, IL-5 and IL-13 levels compared to the levels. Besides, it also decreases the IgE and IgG1 expression	Bui et al. (2019c)
				Blood serum from BALB/c mice		
*Chamaecyparis obtusa* (Siebold and Zucc.) Endl	Cupressaceae	Leaves	Essential oil	*In vivo*	Inhibited the production of IgE, reduces the inflammatory mediators level (IL-4, IL-10, IFN-ƴ, and TNF-α) in NLF. It also inhibits the IL-4, GATA-3 expressions as well as IL-10 and Foxp3 mRNA expressions in sinonasal mucosa	Shin et al. (2020)
				IgE levels, nasal lavage fluid (NLF), splenocytes and sinonasal mucosa of OVA induced BALB/c mice		
*Cinnamomum verum* J.Presl	Lauraceae	Bark	Standardized hydroalcoholic extract	*In vivo*	Prevented the elevation of histamine and IgE levels	Aswar et al. (2015)
				OVA induced male BALB/c mice (histamie challenge based on effects on nasal sign)		
*Cissampelos sympodialis* Eichler	Menispermaceae	Root	Warifteine (**3**) and methylwarifteine (**4**)	*In vivo*	Reduced the number of inflammatory cells, eosinophils, IgE, and cytokines (IL-4, IL-13,1L-5 and Il-17)	Cavalcanti et al. (2020)
				Nasal lavage fluid (NAF) and bronchoaveolar lavage fluids (BALF) of OVA induced isogenic female BALB/c mice (20–25 g)		
—	—	Leaves	Okicamelliaside (**2**) (OCS)	*In vivo*	Suppressing degranulation as it has shown no significant differences in IgE levels	Kuba et al. (2008)
				Blood samples from heart was taken for IgE measurement from Japanese cedar pollen induced male BALB/c mice		
*Citrus deliciosa *Ten	Rutaceae	Fruit	50% methanol extract from MEC (*Citrus unshiu* Powder)	*In vitro*	Inhibited histamine release and decrease the release of ß-hexosaminidase	Kobayashi and Tanabe (2006)
				Basophils of patients with seasonal allergic rhinitis to pollen and rat basophilic leukemia RBL-2H3 cells		
*Cryptomeria japonica*(Thunb. ex L.f.) D.Don	Cupressaceae	Pollen	Transgenic rice containing the hypoallergenic pollen of the plant	*In vivo*	Reduced IgE antibody and peripheral blood mononuclear cell (PBMC) proliferation by inducing the oral immune tolerance against japanese cedar allergens	Saito et al. (2020)
				Blood samples of 4 Japanese monkey with Japanese Cedar Pollinosis and 2 healthy monkey fed with 20 g of raw polished transgenic rice seeds containing destructed Cry j1 and Cry j2 derivatives (10–25 mg antigens/20 g seeds)		
*Eupatorium cannabinum* L	Asteraceae	Arial	60% ethanol extracts	*In vivo*	Inhibited IL-8 and TNFα production in LPS-stimulated human neutrophils	Michalak et al. (2019)
				Lipopolysaccharides (LPS) stimulated human neutrophils		
*Lonicera japonica* thunb	Caprifoliaceae	Flower	95% ethanol extract	*In vivo*	**i**nhibited airway eosinophilia, IgE production and cytokines expression	Bai et al. (2020)
				Nasal septum mucosal tissues		
*Mangifera indica* L	Anacardiaceae	Tree	Mangiferin (**8**)	*In vivo*	Reduced eosinophil, IgE, IgG, histamine levels, IL-4, IL-5, IL-13, IL-17, IL6, GATA-3, RORƴ, TNF α and increase in IFNƴ level	Piao et al., (2020)
				Nasal, lung tissue and nasal lavage fluid of OVA-induced AR model		
*Menth*a × *piperita* L	Lamiaceae	Arial	Luteolin-7-*O*-rutinoside (**5**)	*In vivo*	Protection from mast cell degranulation	Inoue et al. (2002)
				Rat peritoneal mast cells		
*Ostericum grosseserratum* (Maxim.) Kitag	Apiaceae	Root	Methanol Extracts	*In vivo*	Decreased histamine release and have immunosuppressive effects on mast cells and meaningfully inhibited the antigen-induced mRNA expression and production of inflammatory cytokines related to allergic reactions. Also inhibits the IgE production	Jung et al. (2011)
				Blood samples from Male Balb/c mice (10 weeks old)		
*Petasites hybridus* (L.) G.Gaertn., B.Mey. and Schreb	Asteraceae	Leaves	Petasin (**7**)	*In vitro*	Inhibited the IL-8 expression following PolyIC stimulation	Steiert et al. (2017)
				Primary human nasal epithelial cells		
*Phleum pratense* L	Poaceae	Leaves	Aqueous grass pollen allergen extract	*In vitro*	Reduced the production of IL-5	Till et al. (1997)
				Cultures of house dust mite stimulated peripheral blood mononuclear cells from patient with history of severe summer hay fever		
*Piper nigrum* L	Piperaceae	Fruit	70% Ethanol extracts	*In vivo*	Decreased the production of eosinophils, neutrophils and macrophages cells in NALF. Inhibits the phosphorylation of NFκBp65-free andbNFκBp65, IκBα. It also may down regulates the Th17-related cytokines such as factor RORc, IL17A and Th2-related cytokines like IL-5, IL-13 and IL-6	Bui et al. (2019a)
				Nasal lavage fluid (NALF) from Male six-week-old BALB/c mice		
—	—	Fruit	70% Ethanol extracts	*In vivo*	Suppressed the OVA-specific antibodies, serum histamine release and inflammatory cells accumulation. It enhanced the activation of Nrf2/HO-1 signaling	Bui et al. (2020)
				Cell count for nasal lavage fluid, nasal mucosa and blood sample of OVA-induced BALB/c mice		
*Rosa multiflorae* thunb	Rosaceae	—	Standardized extract from *Rosae Multiflorae* extract powder (RMFE)	*In vivo*	Inhibited the accumulation of eosinophils in the nasal mucosa, nasal lavage fluid (NALF) and goblet cells in the nasal epithelium, and mast cells in the respiratory region of the nasal cavity. It also suppressed Th2-related cytokines in NALF, NALT, and splenocytes, whereas the Th1-associated cytokine IL-12 was upregulated by RMFE.	Bui et al. (2019b)
				NALF, nasal tissue, spleen and nasal-associated lymphoidtissue (NALT) from OVA-induced AR mouse model		
*Symphytum officinale* L	Boraginaceae	Root	Shikonin (**1**)	*In vivo*	Decreased Ig-E and IL-4 and GATA-3 expression level. Increse levels of IFN-γ, superoxide dismustase and Malondialdehyde	Wang et al. (2017)
				Nasal mucosa tissue from AR model rats		
*Stephania tetrandra* S.Moore	Menispermaceae	Root	Hot water extract and 99% ethanol extract	*In vivo and in vitro*	Decreased plasma Ig-E concentration and degranulation levels	Maeta et al. (2017)
				Plasma samples of 26 week old BALB/c female mice (*In vivo*)		
				RBL-2H3 cells (*In vitro*)		
*Tussilago farfara* L	Asteraceae	Flower	Tussilagone (**6**)	*In vivo and In vitro*	Reduced the production of IgE, histamine, and IL-6. It also inhibits Lyn/Syk, NF-κB and p38 MAPK signaling pathways in activated mast cells	Jin et al. (2020)
				Nasal mucosa tissue of OVA-sensitized guinea pig (*In vivo*)		
				RBL-2H3 cells (*In vitro*)		
*Xanthium strumarium* L	Asteraceae	Fruit	75% aqueous ethanol extracts	*In vivo*	Inhibited the releases of histamine in bone marrow-derived mast cells. It also decreased the serum levels of IgE, IL-1, IL-4 and IL-5 of AR rats, whereas increased the IFN- level in serum	Peng et al. (2019)
				Nasal mucosa of allergic rhinitis model rats		

**TABLE 2:** Clinical trials studies of plants with anti-allergic rhinitis.

Plant name	Family	Country	Study design	Dosage form	Mechanism of action/Subject	References
*Artemisia abrotanum* L	Asteraceae	Sweden	Open label “proof of concept” study	Nasal spray	The flavonoid fraction in the nasal spray was likely to inhibit the effects mediated by histamine in the nasal mucosa	Remberg et al. (2004)
					12 patients with diagnoses of allergic rhinitis, allergic conjunctivitis and/or bronchial obstructive disease. Only 6 patients were being administered with this preparation without concomitant use of other types of antihistamine	
*Artemisia annua* L	Asteraceae	China	Phase 3, randomized, double-blind, placebo-controlled study	Oral liquid	Used as immunotherapy. Further studies are needed to identify the immunologic mechanisms involved	Lou et al. (2020)
					Patients with AR were randomized into 2 groups at a ratio of 2:1, sublingual immunotherapy group (*n* = 395) and placebo group (*n* = 195)	
*Astragalus mongholicus* Bunge	Fabaceae	Croatia	Double blind, placebo-controlled clinical trial	Oral capsule	Decreased the expression of IgE, IgG and eosinophils	Matkovic et al. (2010)
					48 adult outpatient participants of both sexes with a known history of moderate to severe SAR during the grass (*n* = 26) or weed pollen season (*n* = 22)	
*Betula lenta* L	Betulaceae	United Kingdom	Randomized, placebo-controlled, double-blind, double-dummy study	Birch pollen extract	Acted by influencing basic immunological mechanisms resulting in the suppression of the seasonal increase in eosinophil, in reduction of the late-phase reactivity. It also initiate and maintain the shift from a Th2- to Th1-like response. 89 patients (mean age 30 years, range 20–58 years) with at least 2 years of seasonal birch pollen rhinoconjunctivitis uncontrolled by conventional pharmacotherapy were enrolled	Khinchi et al. (2004)
*Camellia sinensis* (L.) Kuntze	Theaceae	Japan	Open-label, single-dose, randomized, parallel-group study	Tea drink	**S**trongly inhibited mast cell activation through the prevention of tyrosine phosphorylation (Lyn, Syk, and Btk) of cellular protein, myosin light chain phosphorylation, and the expression of FcεRI.	Maeda- Yamamoto et al. (2009)
					38 subjects with Japanese cedar pollinosis. The subjects were randomly assigned to long-term	
*Glycine* max (L.) Merr	Fabaceae	Japan	A randomized, double-blind, placebo-controlled parallel group design	Oral capsules	Inhibited histamine release in animal study	Kobayashi et al. (2004a); Kobayashi et al. (2004b)
					Aged 20–60 years, 24 patients with a well-documented history of PAR for the last 2 years, were enrolled	
*Nepeta bracteata* Benth	Lamiaceae	Iran	A randomized double blind clinical trials	*Nepeta bracteata* Syrup (NBS)	Inhibited Ig-E and have anti-allergic activities such as prevention of the expression of CD40 receptor ligand by basophils and inhibition of the release of histamine by interleukin IL-4 and IL-3	Hajiheydari et al. (2017)
					71 patients completed the trial, 37 patients in treatment, and 34 ones in placebo group	
*Nigella sativa* L	Ranunculaceae	Iran	Prospective and double blind study with descriptive analytic	*N. sativa* oil	Decreased IgE and eosinophil levels	Nikakhlagh et al., (2011)
					66 patients (case and control) with allergic rhinitis (22 males and 44 females)	
*Petasites hybridus* (L.) G.Gaertn., B.Mey. and Scherb	Asteraceae	Switzerland	Randomized, double blind, parallel group comparison	Oral tablet	Inhibited the biosynthesis of leukotrienes, which may be associated with antispasmodic activity and anti-inflammatory action in type I hypersensitivity	Thomet, et al. (2002)
					131 patients were screened for seasonal allergic rhinitis and 125 patients were randomized (butterbur 61; cetirizine 64)	
		United Kingdom	A prospective, randomized, double-blind, double-dummy, 3-arm crossover design	Petasol butenoate complex (Ze 339)	Reduced IL-8 and Leukotriene B_4_ (LTB_4_)	Dumitru et al. (2011)
					18 healthy adults with at least 2-years medical history of moderate-to-severe AR to grass pollen	
*Pinus pinaster* Aiton	Pinaceae	United states	A randomized, double-blind, placebo-controlled exploratory study	Oral tablets	Inhibited expression of proinflammatory cytokine IL-1	Ross (2016)
					39 healthy patients (*n* = 39), male and female, with an age distribution between 18 and 65 years. They were randomized into 2 groups, the Pycnogenol test group (*n* = 19) (Manhattan Drug Company, Hillside, New Jersey) or the placebo group (*n* = 20) Ross, (2016)	
*Rubus chingii* var. *suavissimus*(S.K.Lee)	Rosaceae	Japan	A randomized double blind study, placebo controlled, clinical trial	Oral capsules (ten-Cha extracts)	Mast cell stabilizers to inhibit histamine release	Yonekura et al., 2011
L.T.Lu					47 of the patients were being administered with the oral extracts while another 42 patients were on placebo	
*Urtica dioica* L	Urticaceae	Iran	A randomized double blind study, placebo controlled, clinical trial	Oral tablets	Decreased the production of eosinophil	Bakhshaee (2017)
					AR patients (*n* = 40), 2 groups of 20 patients each (Urtidin tab against Placebo)	
*Vitis vinifera* L	Vitaceae	United states	Double-blind randomized control study	Grapeseed extracts	GSE contains catechin monomers which have been demonstrated to inhibit allergeninduced histamine release in passively sensitized rat peritoneal mast cells	Bernstein et al. (2002)
					Patients with SAR and skin prick test sensitivity to ragweed were randomized to 8 weeks of active treatment or placebo which was begun before the ragweed pollen season	
*Zataria multiflora* Boiss	Lamiaceae	Iran	Double-blind randomized control study	Diluted 20% ZM hydro alcoholic extract	Decreased the expression of IL-7	Ariaee et al. (2018)
					AR patients (*n* = 30), 17 female 13 male	
*Zingiber montanum *(J.Koenig) Link ex A.Dietr	Zingiberaceae	Thailand	Randomized, open-label, threeway crossover study	Oral capsule	Inhibited protein synthesis and phorbol 12-myristate 13-acetate-induced MMP-9 gene expression and in human airway epithelial cells, and suppresses MMP-9 cleavage by house dust mites	Tanticharoenwiwat et al. (2017)
					20 allergic rhinitis patients (14 women and 6 men). There were 12 patients with mild intermittent allergic rhinitis, 5 patients with mild persistent allergic rhinitis and 3 patients with severe persistent allergic rhinitis	

## Pathophysiology of Allergic Rhinitis

RA is a type 1 hypersensitivity reaction which occurs when there are specific triggers from allergens’ exposure including pests, dust mites, some molds and pets. This will bring about the subsequent flow of biochemical and immunological activities leading to the clinical manifestation of the disease. Exposure to allergens may induce IgE antibodies to bind to the high-affinity receptor (FcεRI) on the surface of mast cells, basophils, and antigen-presenting cells and causes sensitization (Gould and Sutton, 2008). The most common symptoms in AR are sneezing, pruritus, and reflex secretory responses. These might be due to the release of histamine which activates H_1_ and H_2_ receptors on mucosal blood vessels and H_1_ receptors on sensory nerve endings leading to vascular engorgement (Togias, 2003). IgE expression and histamine release are the important key features in its pathophysiology as they occur during the early phase of the immunological pathway (Okubo et al., 2017). Pathways involved in the pathophysiology of AR will eventually lead to the manifestations of various common symptoms in AR triggered by the allergen in which the patient is sensitive. Understanding the pathophysiology of AR might in a way help to explain the pathways involved in eliciting the symptoms and thus explained the rational treatment approach indicated.

**FIGURE 1:**
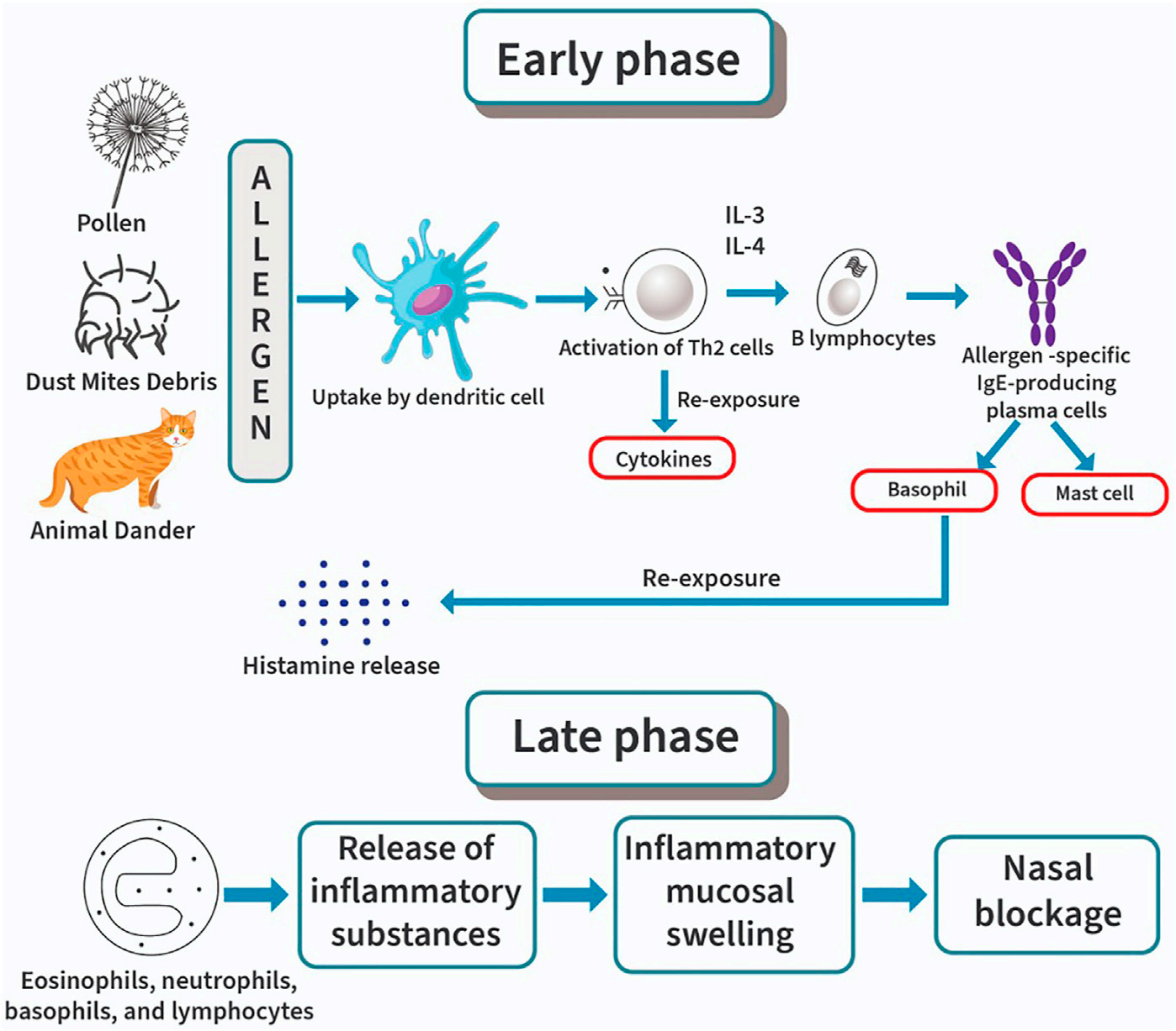
Pathophysiology of allergic rhinitis: early phase and late phase.

Nasal responsiveness occurs with regards to our body normal function or termed as homeostasis in which the body reacts toward an occurrence of abnormal changes with normal response and intensity. Hyperresponsiveness of AR disease might be due to alteration toward the normal responsiveness as a result of genetical or pathological factors affecting the structural or functional elements of the nasal mucosa (Sin and Togias, 2011). AR begins with the allergen sensitization as showed in Figure 1 which leads to the obvious clinical features present in AR patients. Sensitization is a process by which the immune system will produce the antibody or more specifically the IgE antibody, in response to certain types of particles or allergens it considered abnormal. The process begins in the nasal tissues where allergens will be engulfed by antigen-presenting cells (APCs), broken into allergenic peptides and migrate to lymph nodes (Godthelp et al., 1996).

APCs such as immature dendritic cells (DC) capture allergens, mature, and migrate to the draining lymph nodes. The processed allergen will then be presented to naive T cells (Eifan and Durham, 2016). Activation of epithelial cells simultaneously by non-antigenic pathways such as proteases may lead to epithelial cytokines release of which polarizes the action into Th2 response by inducing the expression of genes that promote Th2 survival (Georas et al., 2005). Therefore, Th2 cytokine inhibitors such as suplatast tosilate can be used in managing mild symptoms induced by Th2 response (Tomaki et al., 2000). The release of interleukin-4 (IL-4) may generate Th2 cell production that leads to B-cells activation (Shirakawa et al., 2000). It will become allergen-specific IgE-producing plasma cells that will then bind to mast cells and basophils. This occurrence will eventually lead to repeated AR events as the IgE binding to mast cell and basophils will recognize the presence of allergens invading the body when re-exposure occurred (Wheatley and Togias, 2015).

DC form a network that is localized within the submucosa and epithelium of the entire respiratory mucosa which as well includes the nasal mucosa (Hartmann et al., 2006). In AR patients, there is an elevation in the levels of both T-cells and DC (Sin and Togias, 2011). Processed antigens and the tissue microenvironment may send signals to DC that will then polarize naive T cells into either Th1 or Th2 cells and their respective cytokines (Liu, 2005). The epithelial cells release thymic stromal lymphopoietin and prostaglandin E_2_ which may also send signals to DC and cause such polarization (Bharadwaj et al., 2007). In both nonallergic and allergic individuals there are actually different proportions of allergen specific IL-10–producing Tr1 cells, IL-4–producing effector T cells and CD251 Tregs (Akdis et al., 2004; Maggi et al., 2007). Clinical allergy will develop based on the balance between certain Treg populations and Th2. Cytokines such as IL-3 and IL-4 provides the first essential signal that causes IgE production *via* B cells activation. Besides, these cytokines may as well induce clonal expansion in a case of IgE expressing memory B cells. In the second signal, costimulatory interaction between CD40 ligand on both B-cell surface and T-cell surface will eventually promote activation of B-cell and switch recombination for the production of IgE (Geha, 1992). A cascade of events including early and late phase responses may be triggered by re-exposure to allergens, leading to symptoms of AR characterized by sudden episodes of nasal itching, sneezing, rhinorrhea and nasal congestion (Sih and Mion, 2010). In the late-phase response, fatigue, congestion, malaise, and irritability occur at 6–24 h after exposure to an allergen that involves the influx of inflammatory cells into the nasal mucosa (Rondón et al., 2009; Haenuki et al., 2012). The pathophysiology of AR is diagrammatically presented in Figure 1.

## Anti-allergic Rhinitis Effects of Medicinal Plants and Their Molecular Mechanisms

Medicinal plants and their bioactive metabolites possess different mechanisms of action in combating the mediators of immune system involved in AR inflammatory cascades or allergic reaction pathways. Some of these phytochemicals have anti-allergic properties as well as anti-inflammatory properties which may be useful to treat different types of allergic diseases and symptoms. Several cellular, animal and clinical studies have been performed to evaluate the anti-allergic and anti-inflammatory properties of medicinal plants particularly in treating AR by using AR-induced model or AR patients. There are numerous number of mediators involved in the pathophysiology of AR. These different targeted cells or mediators are important key components for different types of phytochemicals found in many different medicinal plants. The phytochemicals may be effectively acting against the important key components involved and thus alleviating the symptoms in the patient with AR. Anti-allergic rhinitis effects of medicinal plants and their bioactive metabolites may be by suppression of the immune system through suppression of IgE, inhibition of cytokines production, inhibition of histamine release and suppression of eosinophil production.

### Suppression of Immunoglobulin E

IgE is identified as the key molecule that triggers type 1 hypersensitivity reactions such as atopic dermatitis and AR (Platts-Mills, 2001; Johansson et al., 2004). Production of IgE antibodies may be enhanced by the interleukins, particularly IL-4 and IL-3, produced subsequently after the activation of the Th2 cells. This will eventually increase the regulation of eosinophil functions and promote the growth of mast cells (Busse et al., 1995). IgE is an important potential key component targeted at the early stage of hypersensitive reaction as it leads to the activation of other chemical or immunological cascades in later stages. These antibodies, in turn, attached to specific receptors on other resident cells by binding to high-affinity (cεRI) surface receptors on basophils, mast cells or CD23 on B lymphocytes and eosinophils (Presta et al., 1994). IgE has a short half-life and thus having lower concentration compared to other immunoglobulins in the circulation. However, it is extremely bioactive as it binds to high-affinity receptors on the surface of basophils and mast cells. The basophils and mast cells may be highly sensitive to allergens even when the concentration of IgE is very low in the circulation. IgE production and mast cell degranulation is the significant type I allergic responses which cause the subsequent release of histamine and other mediators in an allergic reaction. Following the early phase of allergic reactions, cytokines release including IL-4, IL-5, IL-6, and TNF-α, enhances IgE synthesis. In contrast, IFN-γ, IFN-α and transforming factor (TGF)-β might also inhibit IgE production (Ayoub et al., 2003).

Several cellular and animal studies have been performed to investigate the suppressive effect of medicinal plants on IgE in AR-induced model. The effect of Bupleurum chinense DC. extract on allergic inflammatory responses was conducted in ovalbumin (OVA)-induced AR mouse model by determining the anti-OVA specific IgE, IgG1 and IgG2a in the serum (Bui et al., 2019c). Results showed that at doses of 100 and 200 mg/kg of *B. chinense*, there was a significant (<0.05 and <0.01, respectively) suppression of anti-OVA specific IgE in a dose-dependent pattern. The anti-OVA specific IgG1 in serum was also decreased in a dose-dependent manner which resulted in the inhibition of histamine release. This data indicated that oral administration of *B. chinense* might ameliorate allergic inflammation responses in AR early phase as this particular action mediated by the IgE antibody is the crucial key component that initiates hypersensitivity reaction in AR.

*Cinnamomum verum* J. Presl bark extract was investigated for its potential effect in suppressing IgE levels in an OVA-induced animal model (Aswar et al., 2015). At 10 and 30 μg/kg concentrations, the plant sample significantly prevented elevation of serum IgE levels as compared with AR control mice (*p* < 0.01 and <0.001, respectively). In comparison with the effect of IgE suppression by the plant extract, a positive control treated by xylometazoline showed statistically significant (*p* < 0.001) inhibition of serum IgE level as compared with AR control mice. This study concluded that the extracts from the bark of *C. verum* showed prophylactic potential against OVA-induced model through suppression of IgE as well as histamine release. This potential medicinal plant might be useful in managing AR. IgE may activate the mast cells *via* the high-affinity receptor FcεRI. Therefore, prevention of IgE serum elevation by the extract of *C. verum* indicated stabilization of mast cells as the mechanism of eosinophil recruitment without affecting level of nitric acid.

An *in vivo* study conducted in OVA-induced AR treated with a methanol extract of *Ostericum grosseserratum* (Maxim.) Kitag. roots showed significant inhibition of OVA-specific IgE production in the OVA-sensitized mice IgE (*p* < 0.001) as compared to control mice given OVA alone (Jung et al., 2011). As the release of cytokines IL-4 and IFN-γ has a significant relation with the production of IgE elicited after exposure to an allergen, 100 mg/kg of the methanol extract of *O. grosseserratum* significantly inhibited IL-4 production (*p* < 0.001), compared with OVA-sensitized mice, and enhanced IFN-γ production (*p* < 0.01). These results demonstrated that *O. grosseserratum* extract could inhibit IgE production by the regulation of IL- 4/IFN-γ ratio in allergic responses.

Shikonin **(1)**, a derivative of naphthoquinone, isolated from the roots of *Symphytum officinale* L. was evaluated for its curative effect on IgE production during allergic reaction in an OVA-induced AR rat model. The findings indicated that in three shikonin groups (intraperitoneal injection with 200, 400 and 600 μg/kg of shikonin), the OVA-specific IgE and IL-4 serum levels were significantly decreased and the serum IFN-γ level was markedly increased as compared with the model group. In addition, the shikonin groups also decreased the GATA-3 protein expression level and increased the T-bet protein in nasal mucosa tissue. There were increase of GSH-Px and SOD levels but a decrease in the MDA level as compared to the model group (Wang et al., 2017). The findings suggested that shikonin could alleviate AR in the rat model through suppression of IgE level besides its regulation of GATA-3 and T-bet protein expression in nasal mucosa tissue and anti-oxidative stress effects.

Several clinical investigations have been conducted to verify whether the medicinal plants may target IgE, to suppress its release in patients with AR. Most of the clinical studies conducted were mainly focused on observing the relief of AR symptoms upon their administration. Measurement of IgE serum level was not commonly conducted in clinical studies. One of the clinical trials conducted to observe significant changes in IgE serum level was a double-blind and prospective study on the effect of the oil of *Nigella sativa* L. in 66 AR patients (Nikakhlagh et al., 2011). High level of IgE that was presented in 57.5% of the volunteers (>100 IU/ml) was not always accompanied by allergy symptoms. In fact, severe allergy symptoms have shown in patients with low IgE levels. Allergen-induced rhinitis that causes an up-regulation in the expression of high-affinity receptors reflects more of its true activity rather than the initial concentration of circulating IgE levels in the serum (Yamaguchi et al., 1997). The mean difference of total serum IgE before and after the treatment and placebo groups was statistically significant (*p* = 0.0708) while the differences in IgE before and after a nasal wash in the *N. sativa*-treatment and placebo groups were also statistically significant at *p* = 0.0065 and 0.2800, respectively. However, the average of IgE from the nasal wash in the *N. sativa*-treatment group before and after the treatment in between both study (*p* = 0.0017) and placebo group (*p* = 0.455) was insignificant (Nikakhlagh et al., 2011).

Okicamelliaside (**2**), an ellagic acid glucoside has been isolated from *Camellia japonica* L. It is a highly potent anti-degranulation and has potential to suppress allergic reaction *in vivo* (Onodera et al., 2010). In an *in vivo* study the compound was investigated for its ability to inhibit AR in male BALB/c mice which were stimulated with a Japanese cedar pollen extract and challenged by nasal instillation of the antigen. Intraperitoneal administration of okicamelliaside at 0.2 mg/kg for 24 days resulted in a decrease in the sneezing frequency during the 10 min immediately after the challenge. Okicamelliaside exhibited 12,000 times potency in suppressing sneezing effect compared to ketotifen fumarate, an anti-allergic drug used as control. The sneezing frequency varied widely between individuals in the control, ketotifen fumarate and okicamelliaside groups. However, there was no significant difference in IgE levels, suggesting that KF and okicamelliaside might exert anti-allergic activity by suppressing degranulation instead (Kuba et al., 2008). Okicamelliaside might have a poor activity against AR model but further investigation is needed to help to understand its anti-allergic effect through different cascades involving different key components particularly in AR.

Previous studies on *Cissampelos sympodialis* Eichler has led to the discovery of several alkaloids and their bioactivities (Barbosa-Filho et al., 2000). It was discovered that warifteine (**3**) was the compound responsible for the immunomodulatory and anti-inflammatory effects of the plant on models of inflammation (Costa et al., 2008). Considering the urgency in developing a monotherapy to treat AR, an *in vivo* study has been conducted to analyze the therapeutic potential of warifteine (**3**) and methylwarifteine (**4**) on the combined AR and asthma syndrome model (CARAS) (Cavalcanti et al., 2020). In this study, the treatment with both compounds has shown a significant decrease of OVA-specific IgE at *p* < 0.01. Nuclear factor-kappa B (NF-κB) activation is related to the production of several inflammatory mediators, including cytokines in the inflamed site while the cytokines such as 1L-4,1L-5, and IL-13 are responsible for the allergic-specific IgE production promoting AR symptoms (Schuliga, 2015). In a similar study, the effect of these two compounds was conducted on NF-κB and both compounds have shown a significant decrease in NF-κB (p65) at *p* < 0.05 (Cavalcanti et al., 2020). The isolated compounds found in *C. sympodialis* may have a promising potential as a phytopharmaceutical prototype for allergies but a further study may have to be conducted for its efficacy and safety in a mammal or human model.

### Inhibition of Histamine Release

Allergen exposure against which they are sensitized, may results in nasal symptoms within minutes as there is cross-linking by the allergen of IgE bound to mucosal mast cells (Wheatley and Togias, 2015). The subsequent cascades are the release of histamine, cysteinyl leukotrienes and prostaglandin D2 (Barnes, 2011). Th2 inflammation develops in the nasal mucosa of the AR patient during the next hours, with the participation of a wide array of cytokines and chemokines. This is due to the complex interaction of mast cells, dendritic cells, eosinophils, T cells, epithelial cells, innate lymphoid cells, as well as the basophils (Barnes, 2011; Sin and Togias, 2011). Histamine irritation of the sensory nerve in the nasal mucosa, transmitted to the sneezing center in the brain particularly the medulla oblongata which then leads to sneezing in AR patients (Okubo et al., 2017). It may also cause plasma leakage through its secretion from the nasal glands or acting directly on the nasal mucosa vessels and leads to watery rhinorrhea (Ichikawa et al., 1991; Okubo et al., 2017). As histamine is one of the most important key components in an early phase of AR pathophysiology and gives immediate symptoms within few minutes of allergen exposure, it has been the potential target in managing AR disease in human. This might provide a fast relieve in a patient with AR stimulated by a certain type of allergens.

*In vivo* study on wild grape or *Ampelopsis glandulosa var. brevipedunculata (Maxim.) Momiy*. hot water extract was conducted on phorbol myristate acetate (PMA)-induced or with histamine-induced upregulation of H_1_ receptor messenger ribonucleic acid (mRNA) expression in human epithelial cells (HeLa) (Islam et al., 2018). Stimulation of HeLa cells with PMA or histamine induced significant and transient increase in H1R mRNA with a maximum of 3 h prior to stimulation (Das et al., 2007). There were significant differences at *p* < 0.01 in three different doses of wild grape extract (WGE) (10, 20, 30 μg/ml) compared to PMA-induced HeLa. Besides, there were significant differences at *p* < 0.05; *p* < 0.01 between 20 and 40 μg/ml of WGE, respectively, compared to histamine-induced HeLa. WGE suppressed histamine signaling through the inhibition of histamine-induced upregulation of H1R gene expression. They reported that PKCδ signaling was involved in the expression of the H1R gene. Western-blot analysis suggested that inhibition of phosphorylation of Tyr311 was the underlying mechanism of the suppressive effect of WGE on the up-regulation of H1R gene expression as Tyr311 was crucial for the activation of PKCδ (Islam et al., 2018). WGE showed a strong activity in inhibiting histamine release conducted against AR-induced model and thus might be helpful in alleviating the symptoms in AR. Clinical studies should be conducted in order to assess its safety and efficacy in a human model.

Extract of *Wurfbainia villosa* var. *xanthioides* (Wall. ex Baker) Skornick. and A.D.Poulsen from Zingiberaceae family significantly inhibited systemic allergic reaction and histamine release (Kim et al., 2007; Kim H.-G. et al., 2015). An *in vivo* study showed significant differences in the reduction of histamine release (*p* < 0.05) at the dose of 10–1000 mg/kg compared to 48/80-induced rat peritoneal mast cell (RPMC) histamine release. *W. villosa* extract has shown several actions that were beneficial in alleviating the symptoms in immediate hypersensitivity reaction. The activity of *W. villosa* in inhibiting histamine release was moderate since there was not so much of significant difference when a higher dose of its extract was used. Apart from its anti-allergic rhinitis effect, the ethyl acetate extract of *W. villosa* modulated tumor growth factor-β (TGF-β) related signaling, primarily *via* the Smad2/3 and Smad7 (gene chromosomes) signaling pathways, in bile duct ligation-induced liver fibrosis subject, suggesting that it has antifibrotic effects against cholestatic liver injury (Kim et al., 2019). Other mechanisms suggested that the ethyl acetate extract of *W. villosa* regulated fibrogenic cytokines, especially TGF-β (Lee et al., 2016).

Hydroalcoholic extract of *Cinnamomum verum* J. Presl standardized to type-A procynidines polyphenols (CZ-TAPP) was evaluated for its anti-allergic effect at the dose of 3, 10 and 30 μg/kg in OVA-induced AR in BALB/c mice. At 10 and 30 μg/kg treatment the animals showed significant prevention of elevated serum histamine levels as compared with AR control mice (*p* < 0.01 and *p* < 0.001, respectively). There were induction of rubbing reflex and sneezing in AR control mice as compared to normal mice upon histamine challenge. These elevation in histamine release may results in increased mucus secretion, vascular permeability, edema and contraction of smooth muscle. The treatment with *C. verum* extracts significantly reduced these histamine-induced symptoms. CZ-TAPP has showed a strong inhibition in IgE serum and histamine release as compared with AR control mice while showing reduction of symptoms as well such as sneezing and rubbing (Aswar et al., 2015).

OVA sensitization and challenge in an *in vivo* model led to an increase in histamine and OVA-specific IgE titers in sera, increased IL-4 release in nasal lavage and infiltration of inflammatory cells in the epithelium and sub epithelium of the nasal mucosa (Bahekar et al., 2008). Oral administration of *Ostericum grosseserratum* (Maxim.) Kitag. methanol extract at doses of 0.5 and 1.0 mg/ml significantly inhibited OVA-specific IgE production in the OVA-sensitized mice (*p* < 0.001) as compared to control mice given OVA alone (Jung et al., 2011). Based on this study, *O. grosseserratum* has shown a strong activity in AR-induced model. Another plant that has exhibited histamine suppression effect was *Artemisia abrotanum* L., prepared as a nasal spray preparation. The use of nasal spray preparation has shown a rapid onset of action and relief of nasal symptoms such as congestion, rhinorrhea, and sneezing within a few minutes of application (Remberg et al., 2004). The overall tolerability in all of the twelve patients dealing with a mild to moderate locally appearing stinging sensation was good although it was reported to happen immediately after applying the nasal spray preparation (Remberg et al., 2004). The histamine action was proved by the reduction of early AR symptoms deemed to be strongly related to the early phase cycle of AR pathophysiology. The flavonoids were likely the important compounds in this species that inhibited the effects mediated by histamine (Bergendorff and Sterner, 1995). This study might have shown a good response on its effect in alleviating the symptoms after its application. However, there was no evidence of histamine inhibition that was clearly explained or well conducted in this particular study.

The 50% ethanol extract of peppermint or *Mentha* × *piperita* L. has been reported to inhibit histamine release from peritoneal mast cells of actively sensitized rats (Inoue et al., 2002). The identify the compounds contained in the peppermint extract which leads to such a remarkable result was determined. There were six flavonoid glycosides isolated from the extract and luteolin-7-*O*-rutinoside **5**) was the one that showed a potent inhibitory effect on histamine release from rat peritoneal mast cells. This compound caused a dose-related inhibition of histamine release with a significant effect at 30 and 100 Mµ (*p* < 0.05 and *p* < 0.01, respectively) as well as having inhibitory effects on sneezing and nasal rubbing at the dose of 300 mg/kg (*p* < 0.01). Another study has also been conducted to thoroughly study the structure of this compound and it was found that the catechol structure in the Bring and the C2–C3 double bond in the C ring were essential for the inhibition of the histamine release (Amellal et al., 1985).

### Inhibition of Cytokines Release

Cytokines particularly cysteinyl-leukotrienes were released by mast cells activated by IgE-mediated mechanism in AR (Pawankar and Ra, 1996). In the early phase of IgE-mediated allergic reaction, some types of cytokines such as IL-4 and IL-13 will be released by activated T lymphocytes and will interact with B lymphocytes to induce the synthesis of allergen-specific IgE (Pawankar et al., 2011). Besides histamine, cytokines is also one of the major vasoactive mediators. Some examples of cytokines such as IL-5 and granulocyte/macrophage colony stimulating factor (GM-SCF) may help to overcome programmed cell death of eosinophils by keeping eosinophils alive for several days or even weeks (Plager et al., 1998). Eosinophils synthesize and release cytokines such as IL-3, IL-5 and GM-CSF that play crucial roles in the late phase and on-going allergic inflammation (Yang et al., 1995).

The nasal mucosal tissues of AR has illustrated an elevation in the levels of pro-inflammatory cytokines such as the IL-1, thymic stromal lymphopoietin (TSLP), and TNF-α (Kim H.-Y. et al., 2015). The administration of 10 mg/kg of *Allium hookeri* Thwaites ethanol extract significantly reduced the increased rubs scores and IgE and IL 4 levels in OVA-sensitized mice with significant values at *p* < 0.05. Being a specific inducer of IgE, IL 4 modulates a variety of inflammatory mediators release from immune cells, resulting in increased vascular permeability, increased mucus secretion in the nasal mucosa and inflammatory cells infiltration (Chai et al., 2017). Therefore, cytokine IL-4 is an important key component in alleviating AR symptoms through a downstream of its level in AR patients. The methanol extract of *A. hookeri* at various concentrations of 100, 200, 300 μg/mL has been shown to exhibit anti-inflammatory activity by inhibition of NO and ROS productions (Jang et al., 2017). Besides, it also downregulated NF-κB signaling pathways that consequently reduced proinflammatory mediators. Moreover, it could inhibit adipogenesis by promoting lipolysis by suppressing adipogenic factors expression including controlled amino acid therapy (CAAT)/enhancer binding protein (C/EBP) and lipoprotein lipase (LPL) in 3T3-L1(mouse cell line) adipocytes (Kim et al., 2019).

As discussed earlier, besides decreasing the serum levels of OVA-specific IgE, shikonin (**1**) was also observed to significantly decreased (*p* < 0.05) the serum levels of IL-4 and significantly increased (*p* < 0.05) the serum IFN-γ level (Wang et al., 2017). Previous study has reported that increasing the IFN-γ level and reducing the IL-4 level in the blood could alleviate the pathological lesion of AR in rats (Li et al., 1996). These findings indicated that the mechanism of shikonin attenuated AR may also involved reducing the level of IL-4 and increasing the level of IFN-γ in the body (Wang et al., 2017).

Apart from interleukins, several other types of cytokines that might be involved in the cascades of allergic reaction in AR are interferon-gamma (IFN-ƴ) and retinoid-related orphan receptor gamma t (RORc). An *in vivo* study investigated the possible chemical mediators or key components involved in the pathophysiology of AR such as Th1 related cytokines as well as Th17 related cytokines (Yang et al., 2008). The experiment conducted on OVA-induced mice showed a significant decrease (*p* < 0.05) in the level of Th1 related cytokines such as IL-12 when treated with 100 mg/kg/day of *Piper nigrum* L. extract with a significant increase in IFN-ƴ (*p* < 0.05). The level of IL-12 showed a significant decrease at *p* < 0.001 when treated with a higher dose of *P. nigrum* at 200 mg/kg/day while there was a significant decrease with p< 0.01 in IFN-ƴ level given with the same dose when compared with OVA group. Besides, *P. nigrum* also showed a significant effect on Th17 related cytokines such as IL-5, IL-13, and RORc. The lowest dose of *P. nigrum* (50 mg/kg/day) given to the subject has also shown a significant decrease on IL-5 and IL-13 levels (*p* < 0.01 and *p* < 0.05, respectively). At the dose of 100 mg/kg/day, *P. nigrum* has shown a significant decrease in the levels of IL-5 with *p* < 0.01 and *p* < 0.05 in IL-13 and RORc levels. There’s a significant decrease at *p* < 0.001, *p* < 0.01, *p* < 0.05 in IL-5, RORc, IL-13 levels respectively compared with the OVA group when 200 mg/kg/ml of *P. nigrum* was given to the subject. This shows that *P. nigrum* has a strong activity in reducing IL-5 levels compared to other types of cytokines at 100 mg/kg/day. *P. nigrum* may provide a promising strategy for immunotherapy in airway diseases such as AR as it exhibits several different mechanisms that act against the cascades in AR model to alleviate the symptoms present through a subsequent series of a pathway in the pathophysiology of AR. RORc was shown to regulate Th17 differentiation, but it’s a deficiency, did not completely abolish TH17 related cytokine expression (Yang et al., 2008).

A syrup formulation of *Zataria multiflora* Boiss. was prepared and investigated in a randomized clinical trial conducted in 43 individuals with a history of seasonal AR. A 20% *Z. multiflora* hydroalcoholic extract was diluted to achieve the final concentration of thymol and carvacrol of 20.5 and 2.85 mg/100 ml, respectively. The study investigated the effect of this formulation on the expression of Treg related cytokines IL-17, IL-4 as well as other types of cytokines and TH17 cells. *Z. multiflora* syrup formulation decreased IL-17 expression with a significant difference at (p< 0.05) compared to the control group (Ariaee et al., 2018). IL-7 is a cytokine which produces TH17 cell that plays a major role in fighting extracellular pathogens (Kleinewietfeld and Hafler, 2013). This pro-inflammatory cytokine can upregulate T cell-triggered inflammation and hematopoiesis by stimulating stromal cells to secret other cytokines and growth factors (Wong et al., 2001). IL-17 may play a great role in reducing AR symptoms (Ariaee et al., 2018). The clinical study that has been carried out against *Z. multiflora* has shown a moderate activity in reducing IL-17 against patient with AR. Apart from its anti-allergic rhinitis effect, the erial parts of *Z. multiflora* has an anti-amnesic effect and might improve memory deficit through anticholinesterase activity. Multiple-dose injection of *Z. multiflora* extract could dose-dependently inhibit acetylcholinesterase (AchE) in the brain hippocampus of scopolamine-induced amnesia rats (Sheibani et al., 2019).

Tussilagone (**6**), a sesquiterpene compound isolated from the dried flower buds of *Tussilago farfara* L. has been identified as the major bioactive component of the plant (Jin et al., 2020). IL-6 is a type of cytokine that is also an important key component in the pathophysiology of AR. The aggravation of the inflammatory symptoms may be induced by the mast cells. This is caused by the recruitment of various inflammatory cells to the nasal mucosa by cytokines including IL-6 and TNF-α (Bernstein et al., 2002). Apart from reducing the levels of histamine and OVA-specific IgE in an *in vivo* study, tussilagone administered intraperitoneally at the dose of 50 and 25 mg/kg has shown a significant decrease (p< 0.01) in cytokine IL-6 levels OVA-sensitized guinea pigs. The inhibitory effect of tussilagone at 50 mg/kg was as good as clarityne, the positive control, at the dose of 10 mg/kg (Jin et al., 2020). Another study has also shown a significant decrease in LPS-stimulated production of IL-1β, and IL-6 mRNA through the inhibition of MAPK and NF-ƙB pathways (Choi et al., 2018). The phosphorylation of MAPKs may upregulate the gene expression of multiple inflammatory cytokines by transcription factor activation (Passante and Frankish, 2009). An *in vitro* study conducted on RBL-2H3 cells, a histamine-releasing cell has also shown an obvious suppression of p38 MAPK in a concentration-dependent manner after the treatment with tussilagone (**6**) at the doses of 1, 10, 100, 500 µM with a significant value of p< 0.01 given at the highest dose (500 µM). The inhibition of the MAPK pathway in the mast cells has been identified to be one of the mechanisms to alleviate AR symptoms in this study (Jin et al., 2020).

Petasol butenoate complex, Ze339, a herbal extract from *Petasites hybridus* (L.) G. Gaertn., B. Mey. and Schreb. leaves is known to be effective in treating AR. The use of this medicinal plant has led to decreased local production of IL-8 and LTB4 measured in nasal lining fluid and a faster recovery from nasal obstruction in allergic patients in a placebo-controlled double blind randomized clinical trial (Dumitru et al., 2011). The study has been conducted in 18 subjects with AR to grass pollen has shown a significant decrease in IL-8 and LTB_4_ expression compared to desloratadine (*p* = 0.025 and 0.014, respectively). Petasin (**7**) is a racemic mixture consisting of the isoforms of isopetasin (**7a**) and neopetasin (**7b**). They have been found to be the active components of the extract of this medicinal plant in an *in vitro* study on human nasal epithelial cells (Steiert et al., 2017). This study concluded that petasin decreased the PolyIC-induced IL-8 expression and neutrophil chemotaxis at the dose of 10 μg/ml with significant value of *p* < 0.01. Thus, Ze339 and its constituents, isopetasin and neopetasin are potential candidates for the development of agents for the treatment of immune deviations associated with continuous cytokine-induced inflammation.

### Suppression of Eosinophil Production

Eosinophils and mass cells are the key inflammatory cells in allergic inflammation. Eosinophil infiltration has been considered as the major attribute of AR mucosal inflammation as it is (Minai-Fleminger and Levi-Schaffer, 2009; Wang et al., 2013). In fact eosinophils infiltration is the best marker of allergic inflammation that results in severe inflammation and causes the most severe symptoms (Ciprandi et al., 2005; Gelardi et al., 2008). IgE production is a proinflammatory process driven by cytokines that act against allergens’ exposure through the mucosal infiltration and actions of plasma cells, mast cells, and eosinophils (Skoner, 2001). Production of eosinophil is induced by Th2 cells in the early phase but it might be released during the latent recruitment phase through the release of cytokines and activation of endothelial cells (Baraniuk, 1997). Eosinophils produce several important cytokines such as IL-5 that act to promote eosinophil survival and activation (Tomaki et al., 2000). The eosinophil may as well degranulates and participate in the response to nasal challenge with allergen apart from triggering nasal provocation evidenced by the recovery of major basic protein in nasal lavage fluids (Naclerio et al., 1994).

An overall state of allergic conditions may be determined by the increase in eosinophils in the blood. Eosinophils level was increased in toluene 2,4-diisocyanate (TDI)-induced allergic mice model showing that this is one of the most important key components in understanding AR. Results from this study have shown that the treatment with an oral dose of *Acanthus ilicifolius* L. ethanolic extract (500 mg/kg) and cetrizine (20 mg/kg) could decrease the count of these inflammatory cells as compared to TDI-control (p< 0.05) (Sardar et al., 2018). Symptoms wise, *A. ilicifolius* ethanol extracts significantly suppressed sneezing and nasal score with medium activity with a significant value at p< 0.05 for both responding variables. It may be well explained with the significant suppression of related key components that play important roles in this disease such as eosinophil.

There is an association between the levels of eosinophil and Th17. Both components have been investigated in an *in vivo* study of *Piper nigrum* L. on OVA-induced subject which has a significant increase in eosinophil level with *p* < 0.001. Treatment with ethanol extract of *P. nigrum* at a dose of 50 mg/kg showed a significant decrease (*p* < 0.05) while at the doses of 100 and 200 mg/kg (*p* < 0.01), the extract showed a medium activity against eosinophil suppression compared with the OVA group (Bui et al., 2019a). This study has also been conducted on the effect of dexamethasone in suppressing eosinophil levels with a significant decrease in eosinophil levels at *p* < 0.001 compared to OVA-group. The study emphasized the important key components of eosinophil which are very crucial in most allergic reactions including AR. Thus, suppression of eosinophil may result in the alleviation of AR symptoms.

In another *in vivo* study, eosinophil suppression was found to be significant when treated with a standardized extract of *Rosa multiflora* Thunb. (Bui et al., 2019b). Different doses (100, 200 and 400 mg/kg) of standardized extract of *R. multiflora* were used in this study. At 100 mg/kg the extract showed medium activity with a significant decrease in eosinophil levels (*p* < 0.01) but stronger activity in eosinophil suppression was observed at the doses of 200 and 400 mg/kg (*p* < 0.001) when compared with OVA group. Dexamethasone, used as a positive control also showed a significant difference similar to 200 and 400 mg/kg of *R. multiflorae* extracts compared with the OVA-induced model. Hence, the standardized *R. multiflorae* extracts at the stated doses might have nearly the same efficacy as the present conventional drug, dexamethasone (Bui et al., 2019b). The relative symptomatic effect might be explained by observing the epithelial swelling of OVA-induced subject as eosinophils-derived mediators induced epithelial damage leading to nasal mucosal swelling (Skoner, 2001). A significant difference in amelioration of epithelial swelling was observed in OVA subject treated with 200 and 400 mg/kg of standardized *R. multiflorae* extracts with significant value at *p* < 0.01 while epithelial swelling was ameliorated with a significant decrease of *p* < 0.001 when treated with dexamethasone. Dexamethasone might have a better effect on alleviating the symptoms by ameliorating the epithelial swelling. However, a clinical trial on its effect on human subjects is needed to investigate its effects on the human subject. Yet, this medicinal plant may have a promising future as an alternative treatment for AR.

There was a statistically significant reduction in mean nasal smear eosinophil count observed in a patient administered with Urtidin F. C tablet containing 150 mg of *Urtica dioica* L. The treatment showed medium activity with significant value at *p* < 0.01 compared to pre-treatment while there are no significant differences in control and placebo group (*p* < 0.001 and *p* < 0.1, respectively) (Bakhshaee, 2017). This study was conducted in a randomized double-blind study clinical trial to identify the significant effects of the tablet formulation among AR patients. However, similar effects were demonstrated between the placebo group compared with the treated group based on the Sino-Nasal Outcome Test 22 (SNOT-22) of stinging nettle controlling the symptoms of AR. Therefore, the outcomes of this study showed a weak association between the effect of eosinophil suppression with its symptomatic relief outcomes.

Mangiferin **8**) isolated from *Mangifera indica* L. has showed a significant decrease in eosinophil level compared to the OVA group at *p* < 0.01 given at the dose of 5 and 20 mg/kg. The result showed a similar significant difference in eosinophil level in the animal models treated with 2.5 mg/kg of dexamethasone (Piao et al., 2020). The number of eosinophils, goblet cells, and mast cells infiltrating the nasal mucosa were estimated quantitatively in the histologic sections. It was shown that mangiferin (**8**) at the dose of 20 mg/kg produced a better protective effect on inflammatory cell production compared to 5 mg/kg mangiferin (**8**) (Piao et al., 2020). Mangiferin (**8**) may have the potential to be a better alternative to alleviate AR symptoms than conventional medicines with less side effects. However, the safety and efficacy of this compound may need further research.

## Toxicology

The medicinal plants discussed in this review might have potential to be developed as alternatives to treat symptoms of AR. However, further studies should be carried out to determine their safety level for human use specifically in treating AR. Safety is the most important aspect in developing medicinal or health products. Thus, toxicity studies of the medicinal plants and their bioactive metabolites should be performed and reviewed to identify their potential acute or chronic toxicity that might be encountered upon using them as therapeutic agents for treating allergic diseases such as AR. Some toxicity studies have been conducted for some of these potential medicinal plants.

An acute and chronic toxicity studies on the 95% ethanol extract of *Cinnamomum verum* J. Presl have been conducted by Shah et al. (1998). Thirty-five mice were randomly divided into six *C. verum*-treatment groups and a control and the mice were observed for signs of toxicity and mortality after 24 h. There was some decreased locomotor activity at 3 g/kg dose, suggesting that it might possess narcotic effects at high doses. In a chronic toxicity study, the mice were treated with 100 mg/kg of *C. verum* extract for three weeks. Mortality in the *C. verum* treatment group was statistically insignificant (*p* > 0.05) compared to the control. It was observed that only one male mouse developed inflammation of hind limb which was cleared up during the next few weeks. The condition of the viscera and vital organs weight in the treated animals were normal and comparable to the control except for a decrease in the weight of the liver. There was a decrease in hemoglobin contents base on hematological studies.

The fixed oil of *Nigella sativa* L. was evaluated for its acute toxicity in mice. Oral dose administration of the oil in the mice showed a LD_50_ value of 28.8 ml/kg, while intraperitoneally administration exhibited a LD_50_ value of 2.06 ml/kg (Zaoui et al., 2002). It was observed that oral and intraperitoneal administration of the oil at all doses resulted in behavioral perturbations with immediate agitation, temporary writhing, followed by a quiet attitude period and sedation. Diarrhea was generally observed and the animals died 12 h after oil administration. In a chronic toxicity study, the rats were administered a daily woral dose of 2 ml/kg body wt. for 12 weeks. The leukocyte and platelet counts were observed and showed that there was a significant difference as compared to the control (*p* < 0.01). Besides, the platelet and leukocyte counts were significantly reduced as compared to the control (*p* < 0.01). The compound believed to cause toxicity in *Petasites hybridus* (L.) G. Gaertn., B. Mey. and Schreb. is pyrrolizidine alkaloids (Dreger et al., 2009). The secondary metabolite in the plants known with its hepatotoxic, cardiotoxic, pneumotoxic and nephrotoxic properties provides a defense mechanism against herbivores. Acute toxicity study of *P. hybridus* extract in Wistar rats revealed oral LD_50_ value of ≥2500 mg/kg body weight with 833–1250-fold, and intraperitoneal LD_50_ value of approximately 1000 mg/kg body weight with 333–500-fold higher than the recommended human doses of common butterbur extract (Danesch and Rittinghausen, 2003). Hemorrhagic necrosis, hepatomegaly, and ascites were found to be the signs of acute toxicity. Moreover, hepatic veins obstruction that results in venous-occlusive disease while in chronic poisoning, liver failure due to necrosis, fibrosis, and cirrhosis were some of the effects observed in subacute toxicity testing (Aydın et al., 2013).

The acute toxicity test of aqueous and hexane extracts of *Urtica dioica* L. at the concentrations of 250, 500, 1000, and 2000 mg/kg administered orally in Wistar rats have shown no mortality during 24 h period. No stereotypical toxic symptoms such as ataxia, convulsion, increased diuresis or diarrhea were observed, except the fourth group (2000 mg/kg) which exhibited symptoms like diuresis and diarrhea (Dar et al., 2013). Acute toxicity effects of aqueous and ethanolic extracts of erial parts of *Zataria multiflora* Boiss extracts in mice were conducted. The animals were injected intraperitoneally with various doses of the extracts and the mortality was determined at 48 h after treatment. Based on the LD_50_ values, the ethanolic extract (3.47 g/kg) was more toxic than the aqueous extract (3.85 g/kg) and these extracts are relatively toxic. The maximum non-fatal doses for the aqueous and ethanol extracts were 2.2 and 2 g/kg, respectively (Hosseinzadeh et al., 2000).

## Conclusion

**FIGURE 2:**
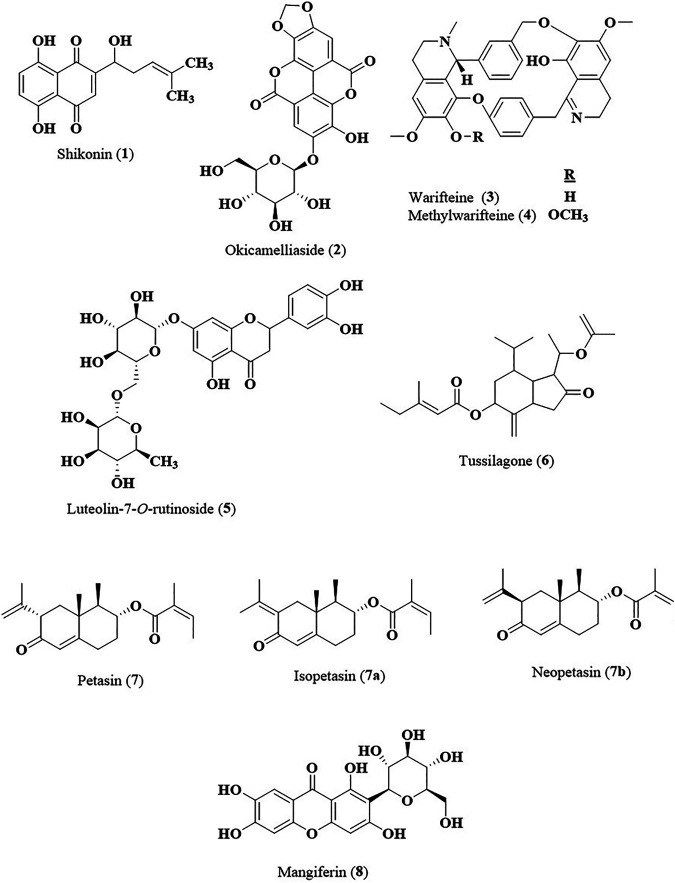
Chemical structures of compounds with strong anti-allergic rhinitis effects.

The most common potential target in treating AR is the suppression of histamine release as histamine is one of the most important key components in an early phase of AR pathophysiology and gives immediate symptoms within few minutes of allergen exposure. Current conventional treatment administers antihistamine drugs as first-line therapy. However, other mechanistic effects such as suppression of IgE, inhibition of cytokines production and suppression of eosinophil production have also been used as targets in efforts to search for bioactive principles from medicinal plants with strong anti-allergic rhinitis activity. Various *in vivo, in vitro* and clinical studies on medicinal plants and their bioactive metabolites have been carried out to evaluate their anti-allergic and anti-inflammatory properties particularly in treating AR by using various immune cells, AR-induced models or AR patients. There was remarkable amount of experimental data that have been generated and they can be further developed as potential source of new anti-allergic rhinitis agents. However, most studies on the medicinal plants including clinical trials were carried out using the crude extracts of the plants as the extracts were not standardized or chemically characterized and the active chemical markers were mostly not identified. The bioactive metabolites contributing to the anti-allergic rhinitis effect have not been well determined. For future studies sufficient preclinical testing should be generated using standardized extracts, which include bioavailability, pharmacokinetic and toxicological studies, before they can be subjected to clinical studies. Based on *in vitro* and *in vivo* studies several bioactive metabolites of the plant extracts including shikonin (**1**), okicamelliaside (**2**), warifteine (**3**), methylwarifteine (**4**), luteolin-7-*O*-rutinoside (**5**), tussilagone (**6**), petasin (**7**), and mangiferin (**8**) (Figure 2) have been identified as potential candidates for development into anti-allergic rhinitis agents. These bioactive compounds have to be further subjected to systematic and operationally thorough controlled randomized trials to prove its safety and efficacy for human use in treating AR.
